# Supplementary material for: Elevated circulating follistatin associates with an increased risk of type 2 diabetes
Source: Nat Commun. 2021 Nov 10;12:6486. doi: 10.1038/s41467-021-26536-w (PMC8580990; doi:10.1038/s41467-021-26536-w)
Supplement: Supplementary file 1 — Supplementary Information [file 41467_2021_26536_MOESM1_ESM.docx]

**Elevated circulating follistatin associates with an increased risk of type 2 diabetes**

**Supplementary Information**

**MDC-CC COHORT**

*Cohort participants*

The Malmö Diet and Cancer study (MDC) is a Swedish population-based, prospective study. During 1991 and 1996, all men and women in the city of Malmö, Sweden, born between 1923 and 1950, were invited to participate in the MDC Cohort ^37,38^. The cardiovascular sub-study (MDC-CC) included a random sample of half of individuals in MDC (n=6103) ^39^. Each participant received a self-administered questionnaire regarding information on current medications as well as on smoking habits at baseline. Blood pressure was measured after 10 min rest in a supine position. Waist circumference was measured midway between the lowest rib margin and iliac crest. Overnight fasting blood samples were collected at baseline. HDL, LDL, blood glucose and hemoglobin A1c (HbA_1c_) were analyzed from blood samples at the local laboratory. Insulin was measured by a radioimmunoassay in mIU/L and the updated Homeostatic Model Assessment (HOMA2) index was calculated (<https://www.dtu.ox.ac.uk/homacalculator>) ^40^. High-sensitivity C-reactive protein (CRP) in plasma was analyzed using the Tina-quant® CRP latex assay (Roche Diagnostics, Basel, Switzerland).

All participants in the study were followed from the baseline examinations until first diagnosis of diabetes, emigration from Sweden, death or end of follow-up (December 31^st^, 2014), whichever came first. Information on incident cases of diabetes in the cohort was collected from six different sources ^41^. Incidence of diabetes was identified using the Malmö HbA_1c_ register (MHR), the regional Diabetes 2000 register, the Swedish National Diabetes Registry (NDR), the Swedish inpatient and outpatient registries and the nationwide Swedish drug prescription registry and the Swedish Cause-of-Death Register. In the MHR, individuals were diagnosed with diabetes if they had at least two HbA_1c_ recordings ≥42 mmol/mol (6.0%) with the Swedish Mono-S standardization system (corresponding to 53 mmol/mol (7.0%)) according to the US National Glycohemoglobin Standardization Program). In the NDR, established criteria (fasting plasma glucose concentration of ≥7.0 mmol/L with two repeated tests on separate occasions) were used to diagnose diabetes. In the Swedish inpatient and outpatient registries, diagnosis of diabetes was made by a physician. In the nationwide prescription registry, diabetes was diagnosed if the person had a filled prescription for insulin or antidiabetic medications (ATC-code A10).

To explore the relationship between follistatin levels in plasma and incidence of diabetes during the follow up, individuals with missing values of covariates, and those with diabetes at baseline and fasting whole blood glucose of >6.1 mmol/L (fasting plasma glucose >7.0 mmol/l) were excluded from the study (Supplement Figure 1). The final study population included 4195 individuals.

Between May 2007 and September 2012, 3734 individuals from the MDC-CC participated in a re-examination. The characteristics of the non-attenders from this examination have been described in detail ^42^. An oral glucose tolerance test (OGTT) after an overnight fast, with a measurement of plasma glucose before and 120 min after intake of 75 g glucose using HemoCue (HemoCue AB, Ängelholm, Sweden) was performed during the reexamination.

**IMI-DIRECT COHORT**

*Cohort participants*

The rationale and design of the epidemiological cohorts within IMI-DIRECT are reported elsewhere ^43^. Individuals with all available baseline follistatin, C-peptide and follow-up (4-year) HbA_1c_ measurements were included in this study, and the sampling frame is from four centers including the METSIM (Kuopio, Finland, Centre 14; n=1079, non-diabetic) ^44^. After excluding participants who did not meet the inclusion criteria (all available variables) or whose data failed quality control, a total of 1074 participants at risk of diabetes in IMI-DIRECT-METSIM were retained.

*Plasma protein biomarker measurements*

HbA_1c_ was measured and documented in patients at baseline and four-year. Plasma glucose, insulin and C-peptide assays were carried out centrally at the University of Eastern Finland (Kuopio, Finland). Glucose was measured by the enzyatic colorimetric assay GOD-PAP using Roche MODULAR P analysers (Hoffmann-La Roche, Basel, Switzerland). Insulin was measured by electrochemiluminescence using Roche E170 Analysers (Hoffmann-La Roche). C-peptide concentrations in plasma were measured by electrochemiluminescence using Roche E170 Analysers (Hoffmann-La Roche). HbA_1c_ was measured by ion-exchange high-performance liquid chromatography using Tosoh G8 analysers (Tosoh Bioscience, San Francisco, CA, USA). Each biochemical assay was performed using validated standard methods. Reference samples were included in all procedures to control for inter-assay variation. Triacylglycerol was measured by quantitative determination with glycerol blanking. HDL-cholesterol was measured directly using polyethylene glycol-modified enzymes and dextran sulphate. When cholesterol esterase and cholesterol oxidase enzymes are modified by polyethylene glycol, they show selective catalytic activity towards lipoprotein fractions, with reactivity increasing in the order LDL < VLDL ≈ chylomicrons < HDL. Total cholesterol was measured by an enzymatic, colorimetric method. LDL-cholesterol was calculated from the total cholesterol, HDL-cholesterol and triacylglycerol concentrations using the Friedewald formula:

LDL=total cholesterol–HDL-cholesterol–(triacylglycerol/2.2)

Cholesterol, glucose, triacylglycerol and HDL-cholesterol were measured using a Roche MODULAR P analyser (Roche Diagnostics, Indianapolis, IN, USA). Insulin and C-peptide were measured using a Roche E170 analyser (Roche Diagnostics). Plasma follistatin levels were measured by the Olink panel as described in the MDC-CC cohort.

**TDFS COHORT**

*Cohort participants*

In the Tübingen Diabetes Family Study (TDFS), data from 210 Caucasians from the southern part of Germany were included in the analyses. They participated in an ongoing study on the pathophysiology of T2D (Stefan N et al., Arch Intern Med. 2008 Aug 11;168(15):1609-16.). Individuals were included into the study when they fulfilled at least one of the following criteria: a family history of T2D, a BMI > 27 kg/m^2^, previous diagnosis of impaired glucose tolerance or gestational diabetes. All subjects had measurements of body fat distribution determined by magnetic resonance imaging. They were considered healthy according to a physical examination and routine laboratory tests. The participants had no history of liver disease and did not consume more than 2 alcoholic drinks per day.

*Oral glucose tolerance test (OGTT)* *and plasma protein biomarker measurements*

All individuals underwent a 75-g oral glucose tolerance test (OGTT). We obtained venous plasma samples at 0, 30, 60, 90, and 120 minutes for determination of plasma glucose and insulin levels. We calculated whole body insulin sensitivity from the OGTT (ISIOGTT) as proposed by Matsuda and DeFronzo ([10,000 / √(Insmean.Glucmean.Ins0.Gluc0)]) ^45^ and with the homeostasis model assessment of insulin resistance (HOMA-IR =Ins0(µU/ml).Gluc0(mmol/l) / 22.5) ^46^. The adipose tissue insulin sensitivity index proposed by Belfiore et al. was calculated (2/[(InsAUC (0, 60, 120).FFAAUC (0, 60, 120)) + 1]) ^47^. Plasma levels of glucose and insulin were measured with the automatic ADVIA 1650 analyzer (Siemens Medical Solutions, Erlangen, Germany). Serum free fatty acid (FFA) concentrations were measured with an enzymatic method (WAKO Chemicals, Neuss, Germany). Fetuin-A was measured with the human fetuin-A Quantikine ELISA (DFTA00, R&D Systems, Minneapolis, MN).

*Body fat mass and liver fat content measurement*

Total body, visceral and subcutaneous fat mass were measured by magnetic resonance (MR) tomography, with an axial T1-weighted fast spin echo technique with a 1.5 T whole body imager (Magnetom Sonata, Siemens Medical Solutions) ^48,49^. Subcutaneous abdominal fat mass and leg fat mass were presented as percentage of total fat mass ^23^. Liver fat content was measured by localized 1H-MR spectroscopy ^48,49^. Nonalcoholic fatty liver disease (NAFLD) was defined as a liver fat content >5.56% ^50^.

**SUMMIT-VIP COHORT**

The SUrrogate markers for Micro- and Macro-vascular hard endpoints for Innovative diabetes Tools-Vascular Imaging Project (SUMMIT-VIP) study cohort was used for replication ^51,52^. Diabetes was defined by current diagnostic criteria (WHO 1998; fasting plasma glucose ≥7.0 mmol/l or 2-h plasma glucose ≥11.1 mmol/l, or both) or by presence of medication with insulin or anti-diabetic drugs. The current study excluded all individuals recruited in Malmö to avoid overlap with the discovery cohort. The current analysis included 885 individuals, 233 with T2D and clinically manifested CVD, 197 with T2D but without clinical signs of CVD, 93 with CVD but no diabetes, 133 individuals without either CVD or diabetes and 229 unreported (T2D or CVD status). Blood samples were genotyped using HumanOmniExpress-12v1_J and HumanOmniExpress-12v1_b chips. Follistatin was measured using a Proseek Multiplex CVD I 96x96 Kit (Olink Bioscience, Uppsala, Sweden) assay as described in MDC-CC cohort.

**EFFECT OF AMG-3969 ON GCK TRANSLOCATION**

The effect of AMG-3969 on GCK translocation was verified in our overexpression system. HepG2 cells were seeded at 3X10^5^ cells/ml on an eight well microscopy chamber (1μ-Slide ibiTreat, Ibidi) and incubated overnight in standard cell culture conditions. Cells were co-transfected with (a) GCK and GCKR expressing plasmids (1:3, GCK:GCKR molar ratio); or (b) GCK with a control vector (1:3; GCK:pCMV6-XL5 molar ratio). Equal molar concentrations of GCK were used for both transfection mixes. Forty-eight hours post-transfection cells were serum-starved in EMEN (5 mM glucose, without antibiotics) for 3 hours, then the medium was changed to the same EMEN with or without AMG-3969 (0.7 μM) and incubated for additional 40 min. The cells were then subjected to standard immunofluorescence procedure. Briefly, cells were washed twice with PBS and fixed in 4% paraformaldehyde (in PBS) for 10 min, washed twice with PBS and then permeabilized with 0.1% PBS-Triton X-100 for 5 minutes. After washing, cells were incubated for 1 hour in blocking solution (1% BSA/10% normal goat serum/0.3M glycine in 0.1%PBS-Tween) at room temperature. GCK primary antibody (5µg/ml in PBS; ab88056, Abcam) was added and the cells were incubated overnight at 4◦C, followed by three washes with PBS for 5 min each before incubating with secondary antibody for 1 hour at room temperature (Goat polyclonal Secondary Antibody to Rabbit IgG - H&L (Alexa Fluor® 488), 1:1000 in PBS; ab150081, Abcam). Finally, the cells were washed twice with PBS and DAPI was added at 0.1 μM in PBS for 20 min at room temperature. Widefield fluorescent images (4 images per condition) were acquire with a Zeiss AXIO Observer. Z1 microscope controlled by Zen imaging software (Zeiss). Fluorescent images were analyzed with Fiji imaging processing software. Two analysis strategies were conducted:

(A) Manual selection of region of interest (ROI) as follows: **1.** conversion of all images to 8-bit images; **2.** background subtraction; **3.** merge channels (green and blue for Alexa-fluor 488 and DAPI, respectively); **4.** manual ROI drawing around the nucleus area (DAPI-positive) of green-positive cells (nuclear-ROI); **5.** manual ROI drawing of the whole the green areas of green-positive cell (whole-green-ROI); **6.** Three main parameters measured: i) area, ii) perimeter and iii) integrated density (total pixel intensity value of the ROI). The measurements were performed over the green images only, whereas the DAPI images were used to segment the nucleus of the cells. We then calculated the % of the nuclear-ROI over the corresponding whole-green-ROI for all three parameters i), ii) and iii). These values represent the % of nuclear-GCK (green) with respect to whole cell GCK signal.

(B) Segmentation-based ROI selection as follows: 1. background subtraction; 2. color threshold segmentation method; 3. measurement of all particles with size equal or bigger than the average nuclear size as calculated from the DAPI images. The same parameters as those in strategy A were measured. A single value was generated for each parameter and compared them between conditions. The smaller the area, perimeter or integrated density, the more nuclear-localized the signal is, indicating higher nuclear localization in the given condition.

**Supplementary Table 1. Baseline characteristics of the MDC-CC based on quartiles of plasma follistatin levels (n=4195)**

|  | **Q1** | **Q2** | **Q3** | **Q4** | ***p*-value** |
| --- | --- | --- | --- | --- | --- |
| **Number (n)** | 1048 | 1049 | 1050 | 1048 |  |
| **Follistatin^†^ (NPX)** | 17.27±1.21 | 23.92±1.07 | 30.06±1.08 | 42.22±1.23 |  |
| **Follistatin men^†^ (NPX)** | 18.13±1.21 | 24.93±1.06 | 31.12±1.07 | 42.52±1.20 |  |
| **Follistatin women^†^(NPX)** | 16.80±1.20 | 23.26±1.06 | 29.45±1.08 | 41.93±1.25 |  |
| **Age (years)** | 55.80±5.84 | 57.22±5.88 | 58.09±5.83 | 58.23±6.01 | < 0.001 |
| **Sex (men) n (%)** | 403(38.5) | 404(38.5) | 404(38.5) | 403(38.5) |  |
| **Waist circumference (cm)** | 80.62±11.62 | 81.78±11.85 | 82.92±12.40 | 84.08±12.41 | < 0.001 |
| **BMI (kg/m^2^)** | 24.76±3.34 | 25.20±3.42 | 25.69±3.87 | 26.00±4.00 | < 0.001 |
| **HDL (mmol/L)** | 1.46±0.38 | 1.41±0.36 | 1.38±0.37 | 1.36±0.37 | < 0.001 |
| **LDL (mmol/L)** | 4.03±0.94 | 4.14 ±0.93 | 4.22±0.99 | 4.27±1.03 | < 0.001 |
| **TG (mmol/L)** | 1.11±0.52 | 1.22±0.56 | 1.31±0.61 | 1.42±0.65 | < 0.001 |
| **CRP mg/L, Median, (P25-P75)*** | 0.80(0.50-1.60) | 1.10(0.60-2.20) | 1.50(0.70-2.90) | 2.00(1.00-4.20) | < 0.001 |
| **Systolic blood pressure (mmHg)** | 135.92±17.44 | 139.31±18.18 | 141.87±19.07 | 143.31±18.84 | < 0.001 |
| **BP lowering medication n(%)** | 122(11.6) | 147(14.0) | 148(14.1) | 184(17.6) | 0.002 |
| **Lipid lowering medication n (%)** | 16(1.5) | 25(2.4) | 20(1.9) | 26(2.5) | 0.385 |
| **Smoking habits n (%)** |  |  |  |  | 0.012 |
| ***Never smokers*** | 437(41.7) | 457(43.6) | 418(39.8) | 412(39.3) |  |
| ***Ex-smokers*** | 397(37.9) | 398(37.9) | 397(37.8) | 71(35.4) |  |
| ***Current smokers*** | 214(20.4) | 194(18.5) | 235(22.4) | 265(25.3) |  |
| **Glucose (mmol/L)** | 4.83±0.44 | 4.89±0.43 | 4.90±0.45 | 4.91±0.45 | < 0.001 |
| **HbA_1c_ (%) (mmol/mol)** | 4.69±0.40 | 4.74±0.40 | 4.82±0.42 | 4.86±0.44 | < 0.001 |
| **HOMA2-IR, Median, (P25–P75)^*^** | 0.80(0.50-1.10) | 0.80  (0.50-1.10) | 0.80  (0.50-1.20) | 0.90  (0.60-1.20) | < 0.001 |
| **HOMA2-B, Median, (P25–P75)^*^** | 63.10  (50.50-76.90) | 64.50  (52.10-79.70) | 66.40  (51.70-83.08) | 68.40  (53.70-86.73) | < 0.001 |
| **Insulin (µU/mL)**  **Median, (P25–P75) ^*^** | 6.00  (4.00-8.00) | 6.00  (4.00-8.00) | 6.00  (4.00-9.00) | 7.00  (5.00-9.00) | < 0.001 |

Median (P25–P75) is presented for CRP, insulin and HOMA2s. All other values are means ± standard deviation or percentages (%). *p* values were calculated using ANOVA (1 degree of freedom) for continuous variables and Pearson’s chi2 for percentages. **p* value for ln transformed value. CRP: C-reactive protein; HOMA: Homeostatic Model Assessment; MDC-CC: Malmö Diet Cancer study cardiovascular cohort. **^†^**Follistatin is expressed as linear Normalized Protein eXpression (NPX) Arbitrary Unit (AU) for relative quantification according to Olink guidance.

**Supplementary Table 2: Correlations of plasma follistatin levels with plasma glucose and insulin levels, HbA_1c_, HOMA2 and glucose levels at 0 and 2 hrs during an oral glucose tolerance test (OGTT) among non-diabetic individuals in MDC-CC**

|  | **Glucose** | **HbA_1c_** | **HOMA2IR** | **HOMA2B** | **Insulin** | **Glucose_0**† | **Glucose_2h**† |
| --- | --- | --- | --- | --- | --- | --- | --- |
| **Model 1** | 0.040** | 0.122*** | 0.099*** | 0.083*** | 0.097*** | 0.054**†† | 0.09***†† |
| **Model 2** | -0.015 | 0.088*** | 0.034* | 0.044** | 0.034* | 0.011††† | 0.061**††† |
| **Model 3** | -0.016 | 0.075*** | 0.027 | 0.038* | 0.027 | 0.003††† | 0.039*††† |

Values are standardized beta coefficients from multiple linear regressions. Model 1: Adjusted for age and sex. Model 2: Adjusted for age, sex, BMI, physical activity, alcohol intake, fiber intake, waist circumference, smoking habits, use of anti-hypertensive medications, systolic blood pressure, LDL, HDL cholesterol, use of lipid lowering medications. Model 3: Model 2 and CRP. Natural log transformed values for HOMA2s, insulin and CRP were used. CRP: C-reactive protein; HOMA2: Homeostatic Model Assessment version 2. **p*<0.05, ***p*<0.01, ****p*<0.001, ­† OGTT after an overnight fast, with a measurement of plasma glucose before and 120 min at re-examination. †† n=2616; ††† n=2568.

**Supplementary Table 3. Baseline characteristics of the IMI-DIRECT-METSIM cohort (Kuopio, Finland; n=1079, non-diabetic) based on quartiles of follistatin**

|  | **Q1** | **Q2** | **Q3** | **Q4** | ***p*-value** |
| --- | --- | --- | --- | --- | --- |
| **Centre** | 14 | 14 | 14 | 14 |  |
| **Number** | 270 | 270 | 269 | 270 |  |
| **Follistatin (NPX)** | 2664.94±343.49 | 3447.24±205.82 | 4194.2±258.59 | 5725.14±1163.54 |  |
| **Age (years)** | 59.99±5.65 | 61±5.53 | 60.84±5.67 | 61.98±5.12 | 0.008 |
| **Sex (men) n (%)** | 270(100) | 270(100) | 269(100) | 270(100) |  |
| **BMI (kg/m^2^)** | 27.23±3.28 | 27.64±3.33 | 28.1±3.67 | 28.04±4.45 | 0.022 |
| **C-peptide (pmol/L)** | 815.74±312.41 | 815.52±254.1 | 832.27±326.14 | 828.04±351.23 | 0.90 |
| **Glucose (mg/dL)** | 104.03±7.98 | 104.28±7.91 | 103.06±8.47 | 101.99±8.4 | 0.005 |
| **Glucose 48-month (mg/dL)** | 108.06±9.22 | 109.24±9.24 | 107.91±9.52 | 108.41±10.3 | 0.38 |
| **Glucose increase (mg/dL)** | 4.03±8.64 | 4.96±7.63 | 4.86±8.07 | 6.42±8.27 | 0.008 |
| **HbA_1c_ (%)** | 5.51±0.25 | 5.53±0.29 | 5.54±0.26 | 5.54±0.29 | 0.34 |
| **HDL (mg/dL)** | 56.23±14.95 | 53.55±12.99 | 52±14.21 | 52.7±15.31 | 0.004 |
| **LDL (mg/dL)** | 111.53±33.84 | 117.91±30.7 | 118.59±34.41 | 121.39±34.6 | 0.006 |
| **TG (mg/dL)** | 110.57±47.67 | 118.4±53.04 | 126.78±60.2 | 136.53±73.26 | 3.24E-06 |
| **CRP (mg/L)** | 0.95±1.16 | 1.56±3.01 | 2.06±4.01 | 2.78±4.69 | 1.50E-08 |

**Supplementary Table 4. Univariate relationships of circulating follistatin with anthropometric and metabolic characteristics in the TDFS cohort (n=210)**

|  | **Follistatin** | |
| --- | --- | --- |
|  | **z** | ***p*** |
| **Sex*** | −0.71 | 0.48 |
|  | ***r*** | ***p*** |
| **Age** | −0.03 | 0.65 |
| **Fasting FFAs** | 0.22 | 0.001 |
| **FFAs minute 60** | 0.28 | <0.0001 |
| **FFAs minute 120** | 0.28 | <0.0001 |
| **Fasting glucose** | −0.03 | 0.69 |
| **Fasting insulin** | 0.15 | 0.03 |
| **HOMA-IR** | 0.14 | 0.04 |
| **IS-Whole-body** | −0.15 | 0.03 |
| **IS-Adipose tissue** | −0.26 | 0.0002 |
| **Fetuin-A** | 0.10 | 0.14 |
| **BMI (kg/m^2^)** | 0.27 | 0.0001 |
| **Total body fat mass_MRT_** | 0.28 | <0.0001 |
| **Visceral fat mass_MRT_** | 0.18 | 0.008 |
| **Percentage subc. abd. fat mass_MRT_** | 0.04 | 0.58 |
| **Percentage subc. leg fat mass_MRT_** | −0.26 | 0.0001 |
| **Muscle mass arms_MRT_** | 0.01 | 0.85 |
| **Muscle mass legs_MRT_** | 0.05 | 0.45 |
| **Liver fat content_1H-MRS_** | 0.23 | 0.0008 |

*Wilcoxon-Test; MRT, magnetic resonance tomography; MRS, magnetic resonance spectroscopy; BMI, body mass index; IS, insulin sensitivity; HOMA-IR, homeostasis model assessment of insulin resistance; FFAs, free fatty acids.

**Supplementary Table 5. Association between circulating follistatin and adipose tissue insulin sensitivity, whole-body insulin sensitivity and liver fat content in multivariate linear regression models**

|  | **IS-Adipose tissue** | | | **IS-Whole-body** | | | **Liver fat content** | | |
| --- | --- | --- | --- | --- | --- | --- | --- | --- | --- |
| **Parameter** | **std. β** | **SE** | ***p*** | **std. β** | **SE** | ***p*** | **std. β** | **SE** | ***p*** |
| **Intercept** | 0 | 0.91 | 0.24 | 0 | 0.89 | 0.87 | 0 | 0.06 | 0.33 |
| **Age** | 0.13 | 0.07 | 0.048 | −0.05 | 0.23 | 0.84 | 0.09 | 0.06 | 0.14 |
| **Sex*** | −0.08 | 0.03 | 0.01 | 0.26 | 0.06 | <0.0001 | −0.34 | 0.06 | <0.0001 |
| **TAT_MRT_** | −0.35 | 0.07 | <0.0001 | −0.40 | 0.07 | <0.0001 | 0.33 | 0.07 | <0.0001 |
| **Follistatin** | −0.17 | 0.07 | 0.009 | −0.06 | 0.06 | 0.38 | 0.17 | 0.07 | 0.009 |

*Female; MRT, magnetic resonance tomography; IS, insulin sensitivity; TAT, total body adipose tissue.

**Supplementary Table 6. Relationships of circulating follistatin with liver fat content, adjusted for major determinants of liver fat content, in multivariate linear regression models**

|  | **Liver fat content** | | | | | | | | | | | | | | |
| --- | --- | --- | --- | --- | --- | --- | --- | --- | --- | --- | --- | --- | --- | --- | --- |
| **Parameter** | ***std. β*** | ***SE*** | ***p*** | ***std. β*** | ***SE*** | ***p*** | ***std. β*** | ***SE*** | ***p*** | ***std. β*** | ***SE*** | ***p*** | ***std. β*** | ***SE*** | ***p*** |
| Intercept | 0 | 0.06 | 0.33 | 0 | 0.06 | 0.57 | 0 | 0.06 | 0.56 | 0 | 0.06 | 0.46 | 0 | 0.06 | 0.37 |
| Age | 0.09 | 0.06 | 0.14 | −0.11 | 0.06 | 0.08 | 0.00 | 0.06 | 0.97 | 0.07 | 0.06 | 0.21 | 0.06 | 0.06 | 0.34 |
| Sex^*^ | −0.34 | 0.06 | <0.0001 | 0.16 | 0.09 | 0.10 | −0.14 | 0.07 | 0.03 | −0.23 | 0.06 | <0.0001 | −0.30 | 0.06 | <0.0001 |
| TAT_MRT_ | 0.33 | 0.07 | <0.0001 | −0.06 | 0.08 | 0.49 | 0.14 | 0.07 | 0.05 | 0.15 | 0.06 | 0.02 | 0.19 | 0.07 | 0.003 |
| **Follistatin** | 0.17 | 0.07 | 0.009 | 0.10 | 0.06 | 0.08 | 0.09 | 0.06 | 0.14 | 0.15 | 0.06 | 0.009 | 0.11 | 0.06 | 0.07 |
| +VAT |  |  |  | 0.73 | 0.11 | <0.0001 |  |  |  |  |  |  |  |  |  |
| +Leg fat mass _(%)_ |  |  |  |  |  |  | −0.44 | 0.07 | <0.0001 |  |  |  |  |  |  |
| +IS-Whole body |  |  |  |  |  |  |  |  |  | −0.43 | 0.06 | <0.0001 |  |  |  |
| +IS-Adipose tissue |  |  |  |  |  |  |  |  |  |  |  |  | −0.38 | 0.06 | <0.0001 |

^*^Female; TAT, total body adipose tissue; VAT, visceral adipose tissue mass; IS, insulin sensitivity.

**Supplementary Table 7. GWAS of plasma follistatin concentrations (Top SNPs) in MDC-CC (non-imputed data, n=4239)**

| **SNP** | **GENE** | **CHR** | **Position** | **EA** | **NEA** | **MAF** | **P_HWE** | **BETA** | **SE** | ***p*** |
| --- | --- | --- | --- | --- | --- | --- | --- | --- | --- | --- |
| rs780094 | *GCKR* | 2 | 27741237 | C | T | 0.3546 | 0.6359 | -0.07897 | 0.01159 | 1.11E-11 |
| rs780093 | *GCKR* | 2 | 27742603 | C | T | 0.3545 | 0.6571 | -0.07805 | 0.0116 | 1.91E-11 |
| exm1435650 | *ZNF333* | 19 | 14805885 | A | G | 0.01403 | 0.6281 | -0.3125 | 0.04647 | 1.98E-11 |
| rs1260326 | *GCKR* | 2 | 27730940 | C | T | 0.3618 | 0.7693 | -0.07703 | 0.01154 | 2.77E-11 |
| exm831645 | *ADAMTS14* | 10 | 72511968 | T | C | 0.01661 | 0.4064 | -0.273000 | 0.04153 | 5.50E-11 |
| exm373967 | *TMEM44* | 3 | 194353889 | A | C | 0.01935 | 0.2711 | -0.2379999 | 0.03961 | 2.05E-09 |
| rs6760250 | *ZNF512* | 2 | 27812252 | A | G | 0.2313 | 0.1687 | 0.07915 | 0.01326 | 2.60E-09 |
| rs1919127 | *C2orf16* | 2 | 27801493 | C | T | 0.2303 | 0.3795 | 0.07722 | 0.01318 | 4.95E-09 |
| exm181843 | *C2orf16* | 2 | 27801759 | G | A | 0.2308 | 0.4006 | 0.07695 | 0.01319 | 5.77E-09 |
| rs4665382 | *NA* | 2 | 27783801 | C | T | 0.2305 | 0.3594 | 0.0764 | 0.01317 | 7.09E-09 |
| exm646262 | *CUX1* | 7 | 101921289 | G | A | 0.04329 | 0.04706 | -0.1542 | 0.02672 | 8.43E-09 |
| exm182139 | *GPN1* | 2 | 27851918 | A | G | 0.2168 | 0.2308 | 0.07612 | 0.0135 | 1.83E-08 |
| exm-rs1260333 | *NA* | 2 | 27748624 | A | G | 0.4162 | 0.8235 | 0.06118 | 0.0112 | 5.00E-08 |
| rs7586601 | *NA* | 2 | 27584666 | G | A | 0.4806 | 0.48 | -0.05277 | 0.01099 | 1.62E-06 |
| rs292858 | *NA* | 20 | 38333579 | G | A | 0.1291 | 0.5466 | 0.07756 | 0.01625 | 1.87E-06 |
| rs12475426 | *SNX17* | 2 | 27599132 | A | G | 0.4299 | 0.4557 | -0.05052 | 0.01105 | 4.94E-06 |
| exm2265307 | *IFT172* | 2 | 27711893 | A | G | 0.4968 | 0.2785 | 0.0494 | 0.01094 | 6.53E-06 |
| rs614673 | *NA* | 3 | 118539108 | T | C | 0.3279 | 0.4607 | 0.05251 | 0.01169 | 7.20E-06 |
| rs2277912 | *FASTKD2* | 2 | 207634236 | G | T | 0.1982 | 0.8646 | 0.0612 | 0.01362 | 7.24E-06 |
| rs17407415 | *NA* | 4 | 131100838 | G | A | 0.3455 | 0.6081 | 0.05164 | 0.01151 | 7.41E-06 |
| rs4804036 | *CCDC9* | 19 | 47767643 | A | G | 0.3225 | 0.01098 | -0.0534 | 0.01195 | 8.08E-06 |
| rs11126999 | *IFT172* | 2 | 27670307 | A | G | 0.4206 | 0.5227 | -0.04944 | 0.01108 | 8.30E-06 |
| rs1260320 | *GCKR* | 2 | 27722416 | A | G | 0.4229 | 0.6772 | -0.04948 | 0.01112 | 8.76E-06 |
| rs4803 | *KRTCAP3,*  *IFT172* | 2 | 27667297 | G | A | 0.4232 | 0.6175 | -0.04913 | 0.0111 | 9.80E-06 |

SNP, single nucleotide polymorphisms; CHR, chromosome; EA/NEA, effect allele/non-effect allele; MAF, minor allele frequency; P_HWE, *p*-value for Hardy-Weinberg equilibrium test; SE, standard error.

**Supplementary Table 8. GWAS and plasma follistatin (Top SNPs) in SUMMIT (imputed data, n=885)**

| RSID | SNP | GENE | NEA | EA | MAF | HWE | INFO | BETA | SE | *p* |
| --- | --- | --- | --- | --- | --- | --- | --- | --- | --- | --- |
| rs1260326 | 2:27730940 | *GCKR* | T | C | 0.435329 | 0.218216 | 0.997982 | -0.296793 | 0.0469133 | 2.51E-10 |
| rs3981860 | 2:114866827 | *DPP10* | C | T | 0.319492 | 0.756994 | 0.996182 | 0.254342 | 0.0506773 | 5.20E-07 |
| rs6057974 | 20:32660765 | *RALY* | T | A | 0.081664 | 1 | 0.467897 | 0.59254 | 0.126585 | 2.86E-06 |
| rs12201018 | 6:102482211 | *GRIK2* | T | C | 0.0552922 | 0.51015 | 0.987458 | -0.496011 | 0.10636 | 3.11E-06 |
| rs9893651 | 17:10185972 | *MYHAS* | C | T | 0.083378 | 1 | 0.998295 | -0.400065 | 0.0859734 | 3.27E-06 |
| rs9606294 | 22:20234525 | *RTN4R* | C | T | 0.137287 | 1 | 0.779888 | 0.362196 | 0.0781004 | 3.53E-06 |
| rs75150178 | 7:46495737 | *IGFBP3* | C | T | 0.0595828 | 0.542218 | 0.860702 | 0.496174 | 0.10704 | 3.56E-06 |
| rs115402209 | 1:179374759 | *AXDND1* | G | T | 0.0564549 | 1 | 0.926719 | -0.490581 | 0.106787 | 4.35E-06 |
| rs7086334 | 10:72310479 | *PALD1* | C | T | 0.431293 | 0.945322 | 0.978616 | -0.221154 | 0.0483179 | 4.72E-06 |
| rs78551615 | 3:76829525 | *ROBO2* | A | C | 0.0605174 | 0.358566 | 0.924857 | -0.479197 | 0.104948 | 4.97E-06 |
| rs9476005 | 6:57329886 | *PRIM2* | C | T | 0.467704 | 0.379812 | 0.999824 | 0.218449 | 0.0482894 | 6.08E-06 |
| rs7832342 | 8:74299242 | *STAU2* | T | C | 0.487472 | 0.420147 | 0.996637 | 0.216905 | 0.0481918 | 6.77E-06 |
| rs686652 | 9:135855572 | *GF11B* | C | T | 0.169554 | 0.718687 | 0.919691 | 0.295586 | 0.0664249 | 8.59E-06 |

SNP, single nucleotide polymorphisms; CHR, chromosome; EA/NEA, effect allele/non-effect allele; MAF, minor allele frequency; HWE, Hardy-Weinberg equilibrium. SE, standard error.

**Supplementary Figure 1.**


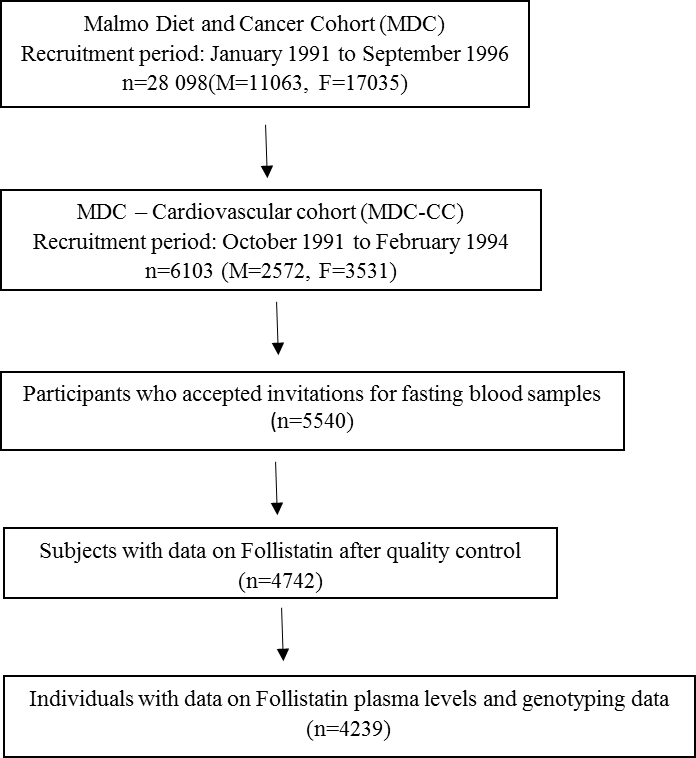


**Supplementary Figure 1. Flow chart of MDC-CC population (n=4239).** For GWAS analysis, all individuals with information on follistatin plasma levels and genotypes (n=4239) were included. The relationship between follistatin levels in plasma and incidence of diabetes during the follow up was explored in 4195 individuals, and fully-adjusted association study was performed in 4060 individuals.

**Supplementary Figure 2.**

**
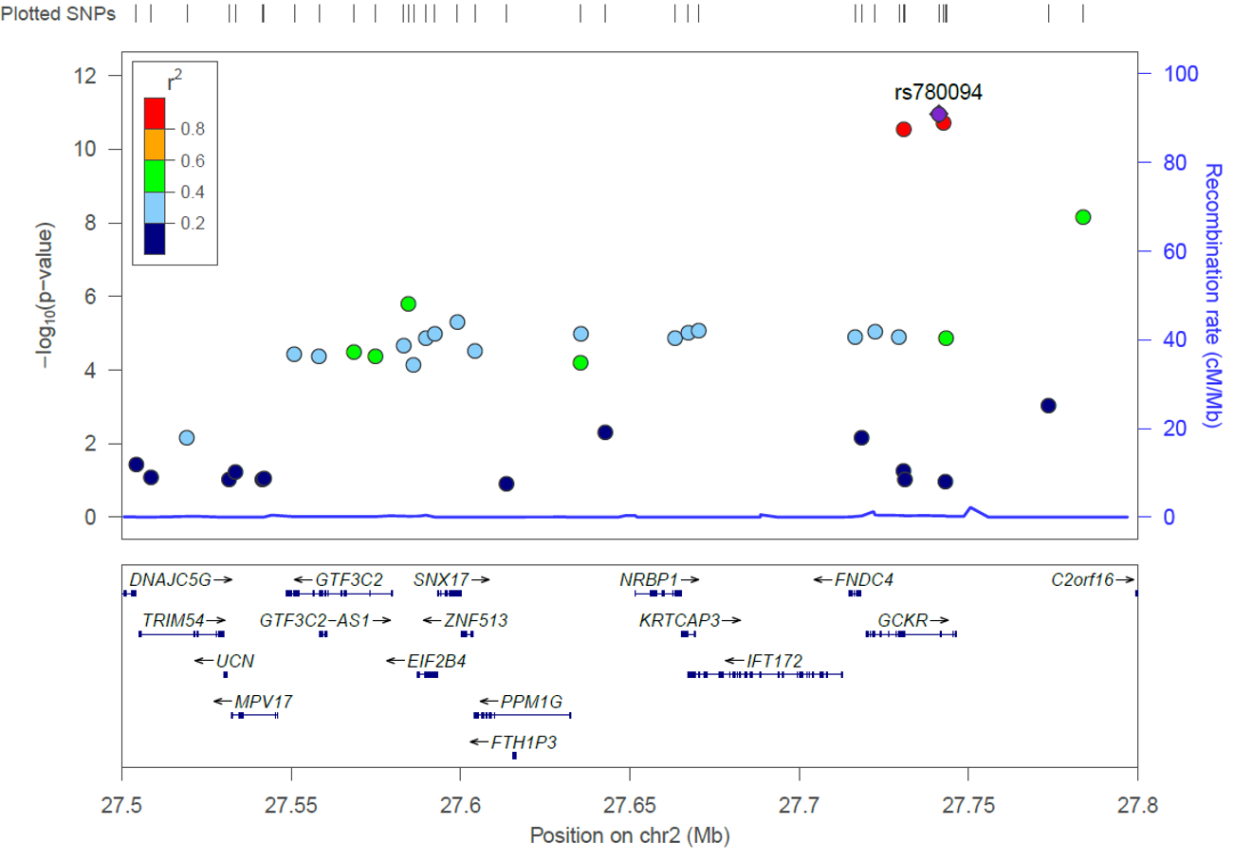
**

**Supplementary Figure 2. Regional plots of chromosome 2 at the *GCKR* locus.**

**Supplementary Figure 3.**
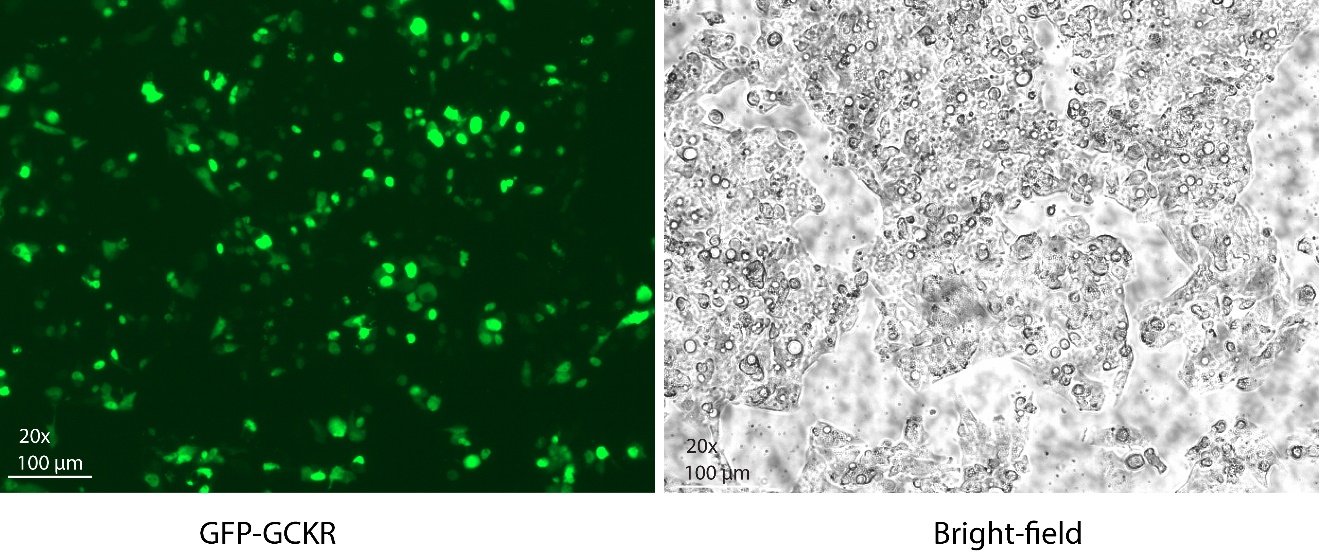


**Supplementary** **Figure 3. Transfection efficiency of GFP-GCKR plasmid in HepG2 cells.** HepG2 cells were transfected with GFP-GCKR (RG214230, Origene) using Lipofectamine 3000 (Thermo Fisher Scientific) according to manufacturer instructions. The cells were imaged 48 h post-transfection in a Zeiss AXIO Observer. Z1 microscope controlled by Zen imaging software (Zeiss). Both fluorescent (left picture) and bright-field (right panel) images were acquired. Images are representative of one experiment performed in 2-3 wells per treatment (n=2-3).

**Supplementary**
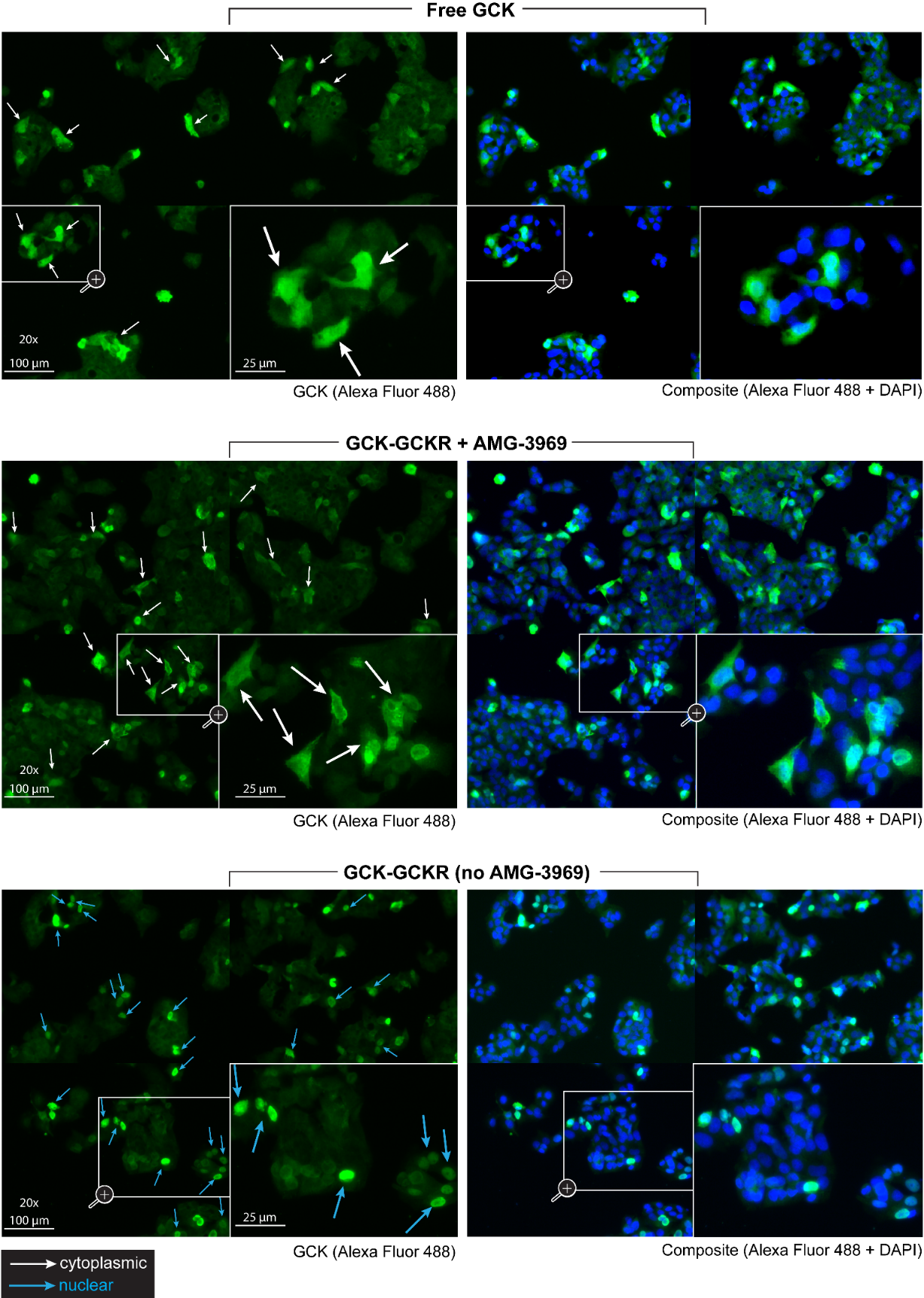
**Figure 4.**

**Supplementary Figure 4. AMG-3669 promoted translocation of disassociated GCK from the nucleus to the cytoplasm in HepG2 cells.** Cellular localization of transfected GCK on fixed HepG2 cells as visualized by fluorescence imaging using a primary polyclonal antibody for human GCK (ab88056). Green fluorescent images (left panels, Alexa Fluor 488) and overlay images (right panels, composite Alexa Fluor 488 + DAPI [nuclear dye]) are shown. Arrows depict cytoplasmic or nuclear localization. Lower right panels are zoomed-in of the selected area in the original image. Images are representative of one experiment performed in 2-3 wells per treatment (n=2-3).

**Supplementary**
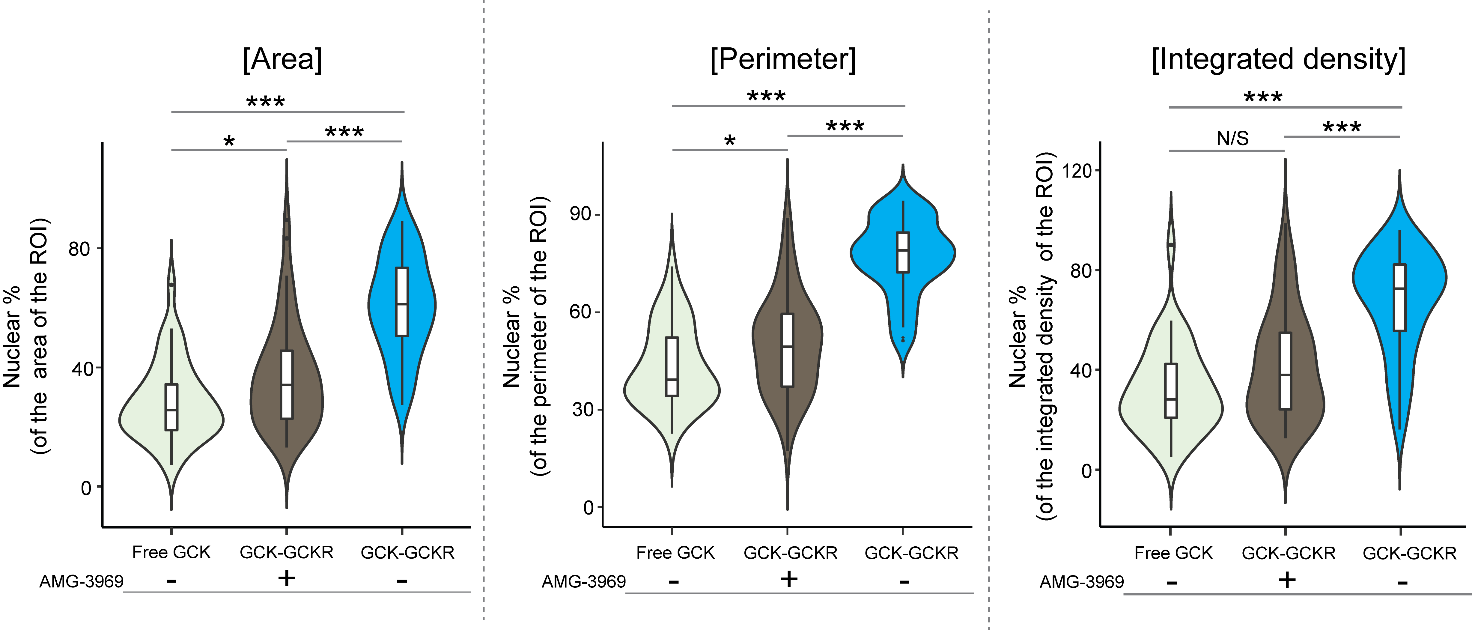
**Figure 5.**

**Supplementary Figure 5.** Violin plots showing the distribution density for the Nuclear % of the Area, Perimeter and Integrated density in each treatment (n=134 cells examined in one experiment comprising 4 non-overlapping images in 2-3 wells per treatment). Box plots show the median (horizontal line in the white box), quartiles (first quartile: white box below the line of the median; third quartile: white box above the line of the median) and the interquartile range (IQR, vertical thin lines). Outlaying values (values > 1.5 x IQR) are plotted as individual points. Treatments were compared with one-way ANOVA followed by a post hoc Tukey HSD test (*p* values, Area: “Free GCK vs GCK-GCKR(+)” *p=*0.04, “Free GCK vs GCK-GCKR(-)” *p=*5.1 x 10^-9^, “GCK-GCKR(+) vs GCK-GCKR(-)” *p=*5.1 x 10^-9^; Perimeter: “Free GCK vs GCK-GCKR(+)” *p=*0.047, “Free GCK vs GCK-GCKR(-)” *p=*5.1 x 10^-9^, “GCK-GCKR(+) vs GCK-GCKR(-)” *p=*5.1 x 10^-9^; Integrated Density: “Free GCK vs GCK-GCKR(+)” *p=*0.072 (N/S), “Free GCK vs GCK-GCKR(-)” *p=*5.1 x 10^-9^, “GCK-GCKR(+) vs GCK-GCKR(-)” *p=*5.7 x 10^-9^).

**REFERENCES**

1 Hansen, J. *et al.* Plasma follistatin is elevated in patients with type 2 diabetes: relationship to hyperglycemia, hyperinsulinemia, and systemic low-grade inflammation. *Diabetes Metab Res Rev* **29**, 463-472, doi:10.1002/dmrr.2415 (2013).

2 Yndestad, A. *et al.* A complex role of activin A in non-alcoholic fatty liver disease. *Am J Gastroenterol* **104**, 2196-2205, doi:10.1038/ajg.2009.318 (2009).

3 Jones, K. L. *et al.* Activin A is a critical component of the inflammatory response, and its binding protein, follistatin, reduces mortality in endotoxemia. *Proc Natl Acad Sci U S A* **104**, 16239-16244, doi:10.1073/pnas.0705971104 (2007).

4 Lee, S. J. Quadrupling muscle mass in mice by targeting TGF-beta signaling pathways. *PLoS One* **2**, e789, doi:10.1371/journal.pone.0000789 (2007).

5 Medeiros, E. F., Phelps, M. P., Fuentes, F. D. & Bradley, T. M. Overexpression of follistatin in trout stimulates increased muscling. *Am J Physiol Regul Integr Comp Physiol* **297**, R235-242, doi:10.1152/ajpregu.91020.2008 (2009).

6 Nakamura, T. *et al.* Activin-binding protein from rat ovary is follistatin. *Science* **247**, 836-838 (1990).

7 Lee, S. J. & McPherron, A. C. Regulation of myostatin activity and muscle growth. *Proc Natl Acad Sci U S A* **98**, 9306-9311, doi:10.1073/pnas.151270098 (2001).

8 Grobet, L. *et al.* A deletion in the bovine myostatin gene causes the double-muscled phenotype in cattle. *Nat Genet* **17**, 71-74, doi:10.1038/ng0997-71 (1997).

9 McPherron, A. C., Lawler, A. M. & Lee, S. J. Regulation of skeletal muscle mass in mice by a new TGF-beta superfamily member. *Nature* **387**, 83-90, doi:10.1038/387083a0 (1997).

10 Zimmers, T. A. *et al.* Induction of cachexia in mice by systemically administered myostatin. *Science* **296**, 1486-1488, doi:10.1126/science.1069525 (2002).

11 Schuelke, M. *et al.* Myostatin mutation associated with gross muscle hypertrophy in a child. *N Engl J Med* **350**, 2682-2688, doi:10.1056/NEJMoa040933 (2004).

12 Braga, M. *et al.* Follistatin promotes adipocyte differentiation, browning, and energy metabolism. *J Lipid Res* **55**, 375-384, doi:10.1194/jlr.M039719 (2014).

13 Hansen, J. S. *et al.* Circulating Follistatin Is Liver-Derived and Regulated by the Glucagon-to-Insulin Ratio. *J Clin Endocrinol Metab* **101**, 550-560, doi:10.1210/jc.2015-3668 (2016).

14 Zhao, C. *et al.* Overcoming Insulin Insufficiency by Forced Follistatin Expression in beta-cells of db/db Mice. *Mol Ther* **23**, 866-874, doi:10.1038/mt.2015.29 (2015).

15 Tao, R. *et al.* Inactivating hepatic follistatin alleviates hyperglycemia. *Nat Med* **24**, 1058-1069, doi:10.1038/s41591-018-0048-0 (2018).

16 Orho-Melander, M. *et al.* Common missense variant in the glucokinase regulatory protein gene is associated with increased plasma triglyceride and C-reactive protein but lower fasting glucose concentrations. *Diabetes* **57**, 3112-3121, doi:10.2337/db08-0516 (2008).

17 Borne, Y. *et al.* Complement C3 Associates With Incidence of Diabetes, but No Evidence of a Causal Relationship. *J Clin Endocrinol Metab* **102**, 4477-4485, doi:10.1210/jc.2017-00948 (2017).

18 Beer, N. L. *et al.* The P446L variant in GCKR associated with fasting plasma glucose and triglyceride levels exerts its effect through increased glucokinase activity in liver. *Hum Mol Genet* **18**, 4081-4088, doi:10.1093/hmg/ddp357 (2009).

19 Rees, M. G. *et al.* Cellular characterisation of the GCKR P446L variant associated with type 2 diabetes risk. *Diabetologia* **55**, 114-122, doi:10.1007/s00125-011-2348-5 (2012).

20 Lopez Rodriguez, M. *et al.* Identification and characterization of a FOXA2-regulated transcriptional enhancer at a type 2 diabetes intronic locus that controls GCKR expression in liver cells. *Genome Med* **9**, 63, doi:10.1186/s13073-017-0453-x (2017).

21 Lloyd, D. J. *et al.* Antidiabetic effects of glucokinase regulatory protein small-molecule disruptors. *Nature* **504**, 437-440, doi:10.1038/nature12724 (2013).

22 Iynedjian, P. B. Molecular physiology of mammalian glucokinase. *Cell Mol Life Sci* **66**, 27-42, doi:10.1007/s00018-008-8322-9 (2009).

23 Stefan, N., Schick, F. & Haring, H. U. Causes, Characteristics, and Consequences of Metabolically Unhealthy Normal Weight in Humans. *Cell Metab* **26**, 292-300, doi:10.1016/j.cmet.2017.07.008 (2017).

24 Karpe, F. & Pinnick, K. E. Biology of upper-body and lower-body adipose tissue--link to whole-body phenotypes. *Nat Rev Endocrinol* **11**, 90-100, doi:10.1038/nrendo.2014.185 (2015).

25 Staehr, P. *et al.* Effects of free fatty acids per se on glucose production, gluconeogenesis, and glycogenolysis. *Diabetes* **52**, 260-267, doi:10.2337/diabetes.52.2.260 (2003).

26 Sakamoto, Y. *et al.* Determination of free follistatin levels in sera of normal subjects and patients with various diseases. *Eur J Endocrinol* **135**, 345-351, doi:10.1530/eje.0.1350345 (1996).

27 Hansen, J. *et al.* Exercise induces a marked increase in plasma follistatin: evidence that follistatin is a contraction-induced hepatokine. *Endocrinology* **152**, 164-171, doi:10.1210/en.2010-0868 (2011).

28 Vamvini, M. T., Aronis, K. N., Chamberland, J. P. & Mantzoros, C. S. Energy deprivation alters in a leptin- and cortisol-independent manner circulating levels of activin A and follistatin but not myostatin in healthy males. *J Clin Endocrinol Metab* **96**, 3416-3423, doi:10.1210/jc.2011-1665 (2011).

29 Raimondo, A., Rees, M. G. & Gloyn, A. L. Glucokinase regulatory protein: complexity at the crossroads of triglyceride and glucose metabolism. *Curr Opin Lipidol* **26**, 88-95, doi:10.1097/MOL.0000000000000155 (2015).

30 Grimsby, J. *et al.* Characterization of glucokinase regulatory protein-deficient mice. *J Biol Chem* **275**, 7826-7831 (2000).

31 Jin, L. *et al.* Role of glucokinase in the subcellular localization of glucokinase regulatory protein. *Int J Mol Sci* **16**, 7377-7393, doi:10.3390/ijms16047377 (2015).

32 Farrelly, D. *et al.* Mice mutant for glucokinase regulatory protein exhibit decreased liver glucokinase: a sequestration mechanism in metabolic regulation. *Proc Natl Acad Sci U S A* **96**, 14511-14516 (1999).

33 Brouwers, M., Jacobs, C., Bast, A., Stehouwer, C. D. A. & Schaper, N. C. Modulation of Glucokinase Regulatory Protein: A Double-Edged Sword? *Trends Mol Med* **21**, 583-594, doi:10.1016/j.molmed.2015.08.004 (2015).

34 Mattisson, I. Y. *et al.* Elevated Markers of Death Receptor-Activated Apoptosis are Associated with Increased Risk for Development of Diabetes and Cardiovascular Disease. *EBioMedicine* **26**, 187-197, doi:10.1016/j.ebiom.2017.11.023 (2017).

35 Assarsson, E. *et al.* Homogenous 96-plex PEA immunoassay exhibiting high sensitivity, specificity, and excellent scalability. *PLoS One* **9**, e95192, doi:10.1371/journal.pone.0095192 (2014).

36 Bao, X. *et al.* Growth differentiation factor 15 is positively associated with incidence of diabetes mellitus: the Malmo Diet and Cancer-Cardiovascular Cohort. *Diabetologia* **62**, 78-86, doi:10.1007/s00125-018-4751-7 (2019).

37 Berglund, G., Elmstahl, S., Janzon, L. & Larsson, S. A. The Malmo Diet and Cancer Study. Design and feasibility. *Journal of internal medicine* **233**, 45-51 (1993).

38 Manjer, J. *et al.* The Malmo Diet and Cancer Study: representativity, cancer incidence and mortality in participants and non-participants. *European journal of cancer prevention : the official journal of the European Cancer Prevention Organisation (ECP)* **10**, 489-499 (2001).

39 Hedblad, B., Nilsson, P., Janzon, L. & Berglund, G. Relation between insulin resistance and carotid intima-media thickness and stenosis in non-diabetic subjects. Results from a cross-sectional study in Malmo, Sweden. *Diabetic Med* **17**, 299-307, doi:DOI 10.1046/j.1464-5491.2000.00280.x (2000).

40 Levy, J. C., Matthews, D. R. & Hermans, M. P. Correct homeostasis model assessment (HOMA) evaluation uses the computer program. *Diabetes care* **21**, 2191-2192 (1998).

41 Enhorning, S. *et al.* Genetic vasopressin 1b receptor variance in overweight and diabetes mellitus. *European journal of endocrinology* **174**, 69-75, doi:10.1530/EJE-15-0781 [doi] (2016).

42 Rosvall, M. *et al.* Risk factors for the progression of carotid intima-media thickness over a 16-year follow-up period: the Malmo Diet and Cancer Study. *Atherosclerosis* **239**, 615-621, doi:10.1016/j.atherosclerosis.2015.01.030 (2015).

43 Koivula, R. W. *et al.* Discovery of biomarkers for glycaemic deterioration before and after the onset of type 2 diabetes: rationale and design of the epidemiological studies within the IMI DIRECT Consortium. *Diabetologia* **57**, 1132-1142, doi:10.1007/s00125-014-3216-x (2014).

44 Stancakova, A. *et al.* Changes in insulin sensitivity and insulin release in relation to glycemia and glucose tolerance in 6,414 Finnish men. *Diabetes* **58**, 1212-1221, doi:10.2337/db08-1607 (2009).

45 Matsuda, M. & DeFronzo, R. A. Insulin sensitivity indices obtained from oral glucose tolerance testing: comparison with the euglycemic insulin clamp. *Diabetes Care* **22**, 1462-1470, doi:10.2337/diacare.22.9.1462 (1999).

46 Matthews, D. R. *et al.* Homeostasis model assessment: insulin resistance and beta-cell function from fasting plasma glucose and insulin concentrations in man. *Diabetologia* **28**, 412-419 (1985).

47 Belfiore, F., Iannello, S. & Volpicelli, G. Insulin sensitivity indices calculated from basal and OGTT-induced insulin, glucose, and FFA levels. *Mol Genet Metab* **63**, 134-141, doi:10.1006/mgme.1997.2658 (1998).

48 Machann, J. *et al.* Follow-up whole-body assessment of adipose tissue compartments during a lifestyle intervention in a large cohort at increased risk for type 2 diabetes. *Radiology* **257**, 353-363, doi:10.1148/radiol.10092284 (2010).

49 Stefan, N. *et al.* Inhibition of 11beta-HSD1 with RO5093151 for non-alcoholic fatty liver disease: a multicentre, randomised, double-blind, placebo-controlled trial. *Lancet Diabetes Endocrinol* **2**, 406-416, doi:10.1016/S2213-8587(13)70170-0 (2014).

50 Szczepaniak, L. S. *et al.* Magnetic resonance spectroscopy to measure hepatic triglyceride content: prevalence of hepatic steatosis in the general population. *Am J Physiol Endocrinol Metab* **288**, E462-468, doi:10.1152/ajpendo.00064.2004 (2005).

51 Shore, A. C. *et al.* Measures of atherosclerotic burden are associated with clinically manifest cardiovascular disease in type 2 diabetes: a European cross-sectional study. *J Intern Med* **278**, 291-302, doi:10.1111/joim.12359 (2015).

52 Goncalves, I. *et al.* Association between renin and atherosclerotic burden in subjects with and without type 2 diabetes. *BMC Cardiovasc Disord* **16**, 171, doi:10.1186/s12872-016-0346-8 (2016).
