# Supplementary material for: Elevated circulating follistatin associates with an increased risk of type 2 diabetes
Source: Nat Commun. 2021 Nov 10;12:6486. doi: 10.1038/s41467-021-26536-w (PMC8580990; doi:10.1038/s41467-021-26536-w)
Supplement: Supplementary file 3 — Reporting Summary [file 41467_2021_26536_MOESM3_ESM.pdf]

nature research

Corresponding author(s): Yang De Marinis

Last updated by author(s): Jul 5, 2021

Reporting Summary

Nature Research wishes to improve the reproducibility of the work that we publish. This form provides structure for consistency and transparency in reporting. For further information on Nature Research policies, see our Editorial Policies and the Editorial Policy Checklist.

Statistics

For all statistical analyses, confirm that the following items are present in the figure legend, table legend, main text, or Methods section.

n/a	Confirmed	
☐	☒	The exact sample size (*n*) for each experimental group/condition, given as a discrete number and unit of measurement
☐	☒	A statement on whether measurements were taken from distinct samples or whether the same sample was measured repeatedly
☐	☒	The statistical test(s) used AND whether they are one- or two-sided<br>*Only common tests should be described solely by name; describe more complex techniques in the Methods section.*
☐	☒	A description of all covariates tested
☐	☒	A description of any assumptions or corrections, such as tests of normality and adjustment for multiple comparisons
☐	☒	A full description of the statistical parameters including central tendency (e.g. means) or other basic estimates (e.g. regression coefficient) AND variation (e.g. standard deviation) or associated estimates of uncertainty (e.g. confidence intervals)
☐	☒	For null hypothesis testing, the test statistic (e.g. *F*, *t*, *r*) with confidence intervals, effect sizes, degrees of freedom and *P* value noted<br>*Give P values as exact values whenever suitable.*
☒	☐	For Bayesian analysis, information on the choice of priors and Markov chain Monte Carlo settings
☐	☒	For hierarchical and complex designs, identification of the appropriate level for tests and full reporting of outcomes
☐	☒	Estimates of effect sizes (e.g. Cohen's *d*, Pearson's *r*), indicating how they were calculated

Our web collection on statistics for biologists contains articles on many of the points above.

Software and code

Policy information about availability of computer code

Data collection	Detailed description on data collection can be found in Supplementary Appendix.
Data analysis	SPSS Statistics (version 359 22); Stata software version 12.0 (Stata Corp, College Station, TX, USA); JMP 13.0.0 (SAS Institute Inc, Cary, North Carolina); R (version 3.5.1); SNPTEST (snptest version 184 2.5.2). The detection of individuals with different ancestry was done by the multidimensional scaling method implanted in PLINK.

For manuscripts utilizing custom algorithms or software that are central to the research but not yet described in published literature, software must be made available to editors and reviewers. We strongly encourage code deposition in a community repository (e.g. GitHub). See the Nature Research guidelines for submitting code & software for further information.

Data

Policy information about availability of data

All manuscripts must include a data availability statement. This statement should provide the following information, where applicable:

- Accession codes, unique identifiers, or web links for publicly available datasets
- A list of figures that have associated raw data
- A description of any restrictions on data availability

All the relevant data supporting the findings of this study are available within this article, in the supplementary material, the source data file, or relevant repositories. For MDC-CC, SUMMIT and TDFS cohorts, Swedish, European and German legislation impose restrictions on public availability of datasets containing pseudonymized information. The full datasets including genome-wide data and phenotypes can be accessed for the MDC-CC through an institutional repository at Lund University (https://www.malmo-kohorter.lu.se/english), and SUMMIT through the SUMMIT vascular imaging project steering committee (jan.nilsson@med.lu.se), and University of Tübingen (Norbert.Stefan@med.uni-tuebingen.de) with pertinent permissions.

Field-specific reporting

Please select the one below that is the best fit for your research. If you are not sure, read the appropriate sections before making your selection.

☒ Life sciences ☐ Behavioural & social sciences ☐ Ecological, evolutionary & environmental sciences

For a reference copy of the document with all sections, see nature.com/documents/nr-reporting-summary-flat.pdf

Life sciences study design

All studies must disclose on these points even when the disclosure is negative.

Sample size	We have studied 4 cohorts: MDC-CC (n=4195); IMI-METSIM (n=1079); TDFS (n=210); SUMMIT-VIP (n=885). Sample based on population-based cohorts from biobanks. All eligible participants with phenotypic data available included in the present analyses.
Data exclusions	In the cohort study, exclusion criteria was based on variable availability. Otherwise no exclusions.
Replication	Epidemiology analysis was replicated in two independent cohorts: MDC-CC (n=4195) and IMI-METSIM (n=1079). GWAS analysis was successfully replicated in two independent cohorts: MDC-CC (n=4195) and SUMMIT-VIP (n=885).
Randomization	Population-based cohorts were used. Potential confounding factors were evaluated in a series of tests and adjusted for potential confounders in regression models.
Blinding	In this study blinding was impossible because exposure can be discovered only by interviewing the study participants, who obviously know whether or not they were a case. At the time point of the collection of the samples used here in the cohort biobanks, study personnel were unaware of the current study and were completely blinded to outcomes and protein concentrations.

Reporting for specific materials, systems and methods

We require information from authors about some types of materials, experimental systems and methods used in many studies. Here, indicate whether each material, system or method listed is relevant to your study. If you are not sure if a list item applies to your research, read the appropriate section before selecting a response.

Materials & experimental systems

n/a	Involved in the study	
☐	☒	Antibodies
☐	☒	Eukaryotic cell lines
☒	☐	Palaeontology and archaeology
☒	☐	Animals and other organisms
☐	☒	Human research participants
☒	☐	Clinical data
☒	☐	Dual use research of concern

Methods

n/a	Involved in the study	
☒	☐	ChIP-seq
☒	☐	Flow cytometry
☒	☐	MRI-based neuroimaging

Antibodies

Antibodies used	In our study, we used Olink panel for protein measurement. A pair of oligonucleotide-labeled antibodies ("probes") are allowed to pair-wise bind to the target protein present in the sample in a homogeneous assay. When the two probes are in close proximity, a new PCR target sequence is formed by a proximity-dependent DNA polymerization event. With Target 96 panels, the resulting sequence is subsequently detected and quantified using standard real-time PCR (www.olink.com).<br>GCK primary antibody (5µg/ml in PBS; ab88056, Abcam); Goat polyclonal Secondary Antibody to Rabbit IgG - H&L (Alexa Fluor® 488), 1:1000 in PBS; ab150081, Abcam.
Validation	We used standard Olink panel in our study. Details on antibody validation can be found in https://www.olink.com/products/olink-explore/assay-validation/

Eukaryotic cell lines

Policy information about cell lines

Cell line source(s)	1. HepG2 were acquired from ATCC. 2. Primary human preadipocytes were acquired from Zenbio (Cat#L020206).
Authentication	1. HepG2 cells have been authenticated by morphological analysis, as compared to the ATCC documentation. 2. For primary human preadipocytes, spec sheet provides donor information.

Mycoplasma contamination	Both cell lines used in the study have been tested negative for mycoplasma contamination.
Commonly misidentified lines (See ICLAC register)	None.

Human research participants

Policy information about studies involving human research participants

Population characteristics	We evaluated the association of circulating follistatin levels at baseline with incident T2D in the MDC-CC cohort. Of 4195 participants (Sex (men) n (%) 1614(38.5); Age (years) 57.33±5.97), 577 (13.75%) individuals developed T2D during a mean (±SD) follow-up time of 19.07 (±5.09) years. We also analyzed an independent cohort, IMI-DIRECT-METSIM (Sex (men) n (%) 1026(100); Age (years) 60.97±5.53). Among 1079 subjects, 53 (4.91%) developed T2D during the follow-up period (4-year). We then investigated relationships of circulating follistatin with adipose tissue insulin resistance and related traits in the TDFS cohort without diabetes (n=210). For the TDFS study, age [years] 44.8±13.3, Sex (% males) 38.1, the participants fulfilled at least one of the following criteria: a family history of T2D, a BMI > 27 kg/m2, previous diagnosis of impaired glucose tolerance or gestational diabetes). We also performed GWAS within the MDC-CC cohort (n=4239). replicated in an independent SUMMIT-VIP cohort: Age (years) 69.4 ± 8.5, Sex (% males) 73.4, 233 with T2D and clinically manifested CVD, 197 with T2D but without clinical signs of CVD, 93 with CVD but no diabetes, 133 individuals without either CVD or diabetes and 229 unreported (T2D or CVD status). (n=885).
Recruitment	The Malmö Diet and Cancer study (MDC) is a Swedish population-based, prospective study. During 1991 and 1996, all men and women in the city of Malmö, Sweden, born between 1923 and 1950, were invited to participate in the MDC Cohort. . For the IMI-DIRECT-METSIM study, individuals with all available baseline follistatin, C-peptide and follow-up (4-year) HbA1c measurements were included in this study, and the sampling frame is from four centers including the METSIM (Kuopio, Finland, Centre 14; n=1079, non-diabetic). For the TDFS study, Individuals were included when they fulfilled at least one of the following criteria: a family history of T2D, a BMI > 27 kg/m2, previous diagnosis of impaired glucose tolerance or gestational diabetes). For the SUMMIT cohort, analysis included 885 individuals, 233 with T2D and clinically manifested CVD, 197 with T2D but without clinical signs of CVD, 93 with CVD but no diabetes, 133 individuals without either CVD or diabetes and 229 unreported (T2D or CVD status).
Ethics oversight	The study was performed in accordance with the Declaration of Helsinki. The ethics committee at Lund University approved the MDC; and SUMMIT study was approved by ethics committees in each of the centres in Malmö (Sweden), Pisa (Italy), Dundee (U.K.), and Exeter (U.K.). For DIRECT-METSIM cohort, approval for the study protocol was obtained from the Ethics Committee of the University of Eastern Finland and Kuopio University Hospital and all participants provided written informed consent at enrolment. The research conformed to the ethical principles for medical research involving human participants outlined in the declaration of Helsinki. For TDFS cohort, informed written consent was obtained from all participants and the Medical Ethics Committee of the University of Tübingen had approved the protocol.

Note that full information on the approval of the study protocol must also be provided in the manuscript.
